# Membership in the Council of Teaching Hospitals and Health Systems Among Emergency Medicine Residency Program–Sponsoring Institutions, 2001-2020

**DOI:** 10.1001/jamanetworkopen.2023.12457

**Published:** 2023-05-09

**Authors:** Christopher L. Bennett, Krislyn M. Boggs, Cameron J. Gettel, Wendy W. Sun, Arjun K. Venkatesh, Carlos A. Camargo

**Affiliations:** 1 Department of Emergency Medicine, Stanford University School of Medicine, Stanford, California; 2 Department of Emergency Medicine, Massachusetts General Hospital and Harvard Medical School, Boston; 3 Department of Emergency Medicine, Yale University School of Medicine, New Haven, Connecticut

## Abstract

This cross-sectional study identifies US institutions sponsoring residency programs and examines whether Council of Teaching Hospitals and Health Systems membership is associated with institution characteristics.

## Introduction

The number of US residency programs has increased in recent years.^1^ Part of this is attributed to the single accreditation system^1^ and an increase in the number of residencies sponsored by for-profit hospitals.^2^ Less is known about the number of these new programs with Council of Teaching Hospitals and Health Systems (COTH) membership or their ability to meet Accreditation Council for Graduate Medical Education (ACGME) program requirements.^3^ This report identifies institutions sponsoring residencies and analyzes trends and characteristics by COTH membership status; focus was placed on emergency medicine residencies given this specialty’s high rate of growth.

## Methods

We performed cross-sectional analyses of public information on emergency medicine residencies from the Society for Academic Emergency Medicine (SAEM)^4^ and COTH member institutions for multiple years between 2001 and 2020.^4^ SAEM information was used given an absence of year-matched ACGME data. Given COTH membership requirements, membership was used to show differences in program attributes^3^; COTH member institutions are required to sponsor, or significantly participate in, at least 4 residency programs, of which at least 2 should be in medicine, surgery, obstetrics and gynecology, pediatrics, family practice, or psychiatry.^3^ Each SAEM-listed residency and COTH member institution was linked to an emergency department using the National Emergency Department Inventory database,^5^ a database of nonfederal, nonspecialty US emergency departments used to describe and compare emergency department characteristics and total visit volumes. Given the effects of COVID-19 on emergency department visit volumes, a 2019 pre-COVID comparator was included.^5^ Given the single accreditation system, a sensitivity analysis excluding programs formerly accredited by the American Osteopathic Association was also completed. Statistics were generated using Stata version 15.1 (StataCorp); given multiple testing, a *P* < .01 was used to indicate significance. This study was approved by the Mass General Brigham institutional review board and followed the Strengthening the Reporting of Observational Studies in Epidemiology (STROBE) reporting guidelines.

## Results

Emergency medicine residencies sponsored by COTH member institutions had higher total visit volumes in both 2019 (130 institutions [98%] vs 80 institutions [97%]) and 2020 (124 [94%] vs 73 [79%]) (Table). COTH member institutions were also more often designated trauma (117 institutions [89%] vs 65 institutions [71%]) and burn centers (36 [27%] vs 9 [10%]). From 2001 to 2020, the number of emergency medicine residency sponsors increased from 108 to 224 while the number and proportion of these sponsors not affiliated with a COTH member institution rose from 14 (13%) to 92 (41%) (Figure). Findings from our sensitivity analysis were similar.

**Table.** Characteristics of Institutions Sponsoring Emergency Medicine Residencies in 2020, Stratified by COTH Membership Status^a^

Characteristic	COTH membership, No. (%)^a^		*P* value
	Yes	No	
Total 2019 ED visit volume^b^			
<20 000	0	1 (1)	.002
20 000-29 999	0	1 (1)	
30 000-39 999	2 (2)	10 (11)	
≥40 000	130 (98)	80 (97)	
Total 2020 ED visit volume^b^			
<20,000	1 (1)	2 (2)	.005
20,000-29,999	1 (1)	5 (5)	
30,000-39,999	6 (5)	12 (13)	
≥40,000	124 (94)	73 (79)	
Child ED visit volume			
<1000	19 (14)	3 (3)	<.001
1000-4999	27 (20)	26 (28)	
5000-9999	19 (14)	27 (29)	
≥10,000	60 (45)	26 (28)	
Unknown	7 (5)	10 (11)	
Region			
Northeast	40 (30)	27 (29)	.62
Midwest	33 (25)	30 (33)	
South	42 (32)	24 (26)	
West	17 (13)	11 (12)	
Adult trauma center			
Yes	117 (89)	65 (71)	.001
No	15 (11)	27 (29)	
Pediatric trauma center			
Yes	58 (44)	12 (13)	<.001
No	74 (56)	80 (87)	
PCI center			
Yes	97 (73)	59 (64)	.13
No	35 (27)	33 (36)	
Stroke center			
Yes	128 (97)	83 (90)	.03
No	4 (3)	9 (10)	
Adult burn center			
Yes	36 (27)	9 (10)	.001
No	96 (73)	83 (90)	
Pediatric burn center			
Yes	25 (19)	2 (2)	<.001
No	107 (81)	90 (98)	

Abbreviations: COTH, Council of Teaching Hospitals and Health Systems; ED, emergency department; PCI, percutaneous coronary intervention.

^a^ National Emergency Department Inventory–USA data cut on April 27, 2022.

^b^ Given changes in total ED-visit volumes associated with the COVID-19 pandemic, a pre-COVID comparator (2019) is included.

**Figure.**
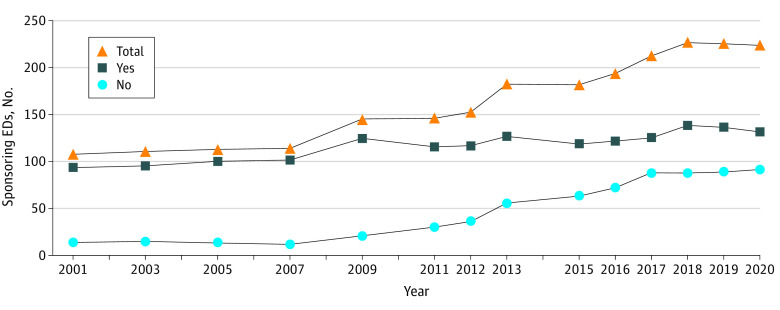
Institutions Sponsoring Emergency Medicine Residencies, Stratified by COTH Membership Status Over Time Abbreviations: COTH, Council of Teaching Hospitals and Health Systems; ED, emergency department.

## Discussion

Among institutions that sponsor emergency medicine residencies in the SAEM database, the proportion without COTH membership increased more than 3-fold since 2001. Visit volumes and institution characteristics also differed by COTH status. Both findings are in the context of the ACGME requiring emergency medicine programs to ensure adequate resources for resident education; this includes the presence of residents in other specialties and their sponsoring site (along with all other emergency department sites, in which residents rotate for 4 months or longer) to have a minimum of 30 000 emergency department visits annually.^6^ Importantly, COTH membership was incorporated as a lens to highlight program differences; absence of membership does not imply the absence of any (or all) other non–emergency medicine residency programs. However, the identified trend and differences by COTH membership status do raise concerns about the educational environment for emergency medicine residents in this growing proportion of programs and the ability of these programs to meet ACGME requirements.^6^ Study limitations stem from our reliance on SAEM data and our use of COTH membership status to highlight differences in program attributes. Identifying the specific resources to each emergency medicine program, the reasons underlying an increasing proportion of sponsoring sites are not in COTH members, or differences by for-profit status was beyond the scope of this work but merit further investigation.

## Notes

Supplement.
Data Sharing Statement
