# Supplementary material for: Membership in the Council of Teaching Hospitals and Health Systems Among Emergency Medicine Residency Program–Sponsoring Institutions, 2001-2020
Source: JAMA Netw Open. 2023 May 9;6(5):e2312457. doi: 10.1001/jamanetworkopen.2023.12457 (PMC10170334; doi:10.1001/jamanetworkopen.2023.12457)
Supplement: Supplement. — Data Sharing Statement [file jamanetwopen-e2312457-s001.pdf]

Data Sharing Statement

Bennett. Membership in the Council of Teaching Hospitals and Health Systems Among Emergency Medicine Residency Program-Sponsoring Institutions, 2001-2020. *JAMA Netw Open*. Published May 09, 2023. doi:10.1001/jamanetworkopen.2023.12457

Data
Data available: No

Additional Information
Explanation for why data not available: All of the datasets (with the exception of the National Emergency Department Inventory) are already public.
